# Expression and clinical significance of inhibitory receptor Leukocyte-associated immunoglobulin-like receptor-1 on peripheral blood T cells of chronic hepatitis B patients: A cross-sectional study

**DOI:** 10.1097/MD.0000000000026667

**Published:** 2021-07-23

**Authors:** Yurong Gu, Yanhua Bi, Huan Wei, Jing Li, Zexuan Huang, Chunhong Liao, Weixin Liao, Yuehua Huang

**Affiliations:** a Guangdong Provincial Key Laboratory of Liver Disease Research, The Third Affiliated Hospital of Sun Yat-sen University, Guangzhou, China; b Department of Infectious Diseases, the Third Affiliated Hospital of Sun Yat-sen University, Guangzhou, China.

**Keywords:** chronic hepatitis B, leukocyte-associated immunoglobulin-like receptor-1, T cell

## Abstract

Leukocyte-associated immunoglobulin-like receptor-1 (LAIR-1) is an inhibitory receptor that is expressed on the surface of multiple immune cells and plays key roles in immune modulation. In patients with chronic hepatitis B (CHB), T cell number and functions are abnormal and the expression of inhibitory receptors is elevated. However, the expression of LAIR-1 on T cells in patients with CHB is still undetermined.

We recruited 320 patients with CHB in different disease phases and 17 healthy donors. Serum biochemical and virological examinations were performed for each participant, and their demographic and clinical data were collected. According to the latest American Association for the Study of Liver Disease guidelines, we categorized the patients into 4 groups: immune active, immune tolerant, inactive CHB, and gray zone. Additionally, we tested the expression of LAIR-1 on T cells and T cell subsets using flow cytometry.

We observed a significant decrease in LAIR-1 expression on CD3+ T cells and its two subsets (CD4+ and CD8+ T cells) in patients with CHB. LAIR-1 expression on T cells was the lowest in the immune active group. LAIR-1 expression levels on CD4+ and CD8+ T cells showed a significant negative association with hepatitis B virus (HBV) DNA load and were lower in hepatitis B e antigen (HBeAg)-positive patients than in HBeAg-negative patients (*P* < .05). In addition, LAIR-1 expression levels on CD3+, CD4+, and CD8+ T cells were all negatively associated with liver inflammation and fibrosis parameters, such as alanine aminotransferase and aspartate aminotransferase levels, FibroScan value, and aspartate aminotransferase-to-platelet ratio index score.

LAIR-1 expression levels on T cells were associated with HBV DNA load and liver inflammation and fibrosis parameters, indicating that LAIR-1 may play an important regulatory role in HBV-induced T cell immune pathogenesis and may be a therapeutic target for CHB.

## 1 Introduction

Chronic hepatitis B (CHB) infection remains a major health problem worldwide. It is estimated that approximately 2 billion people have been infected with the hepatitis B virus (HBV) and >350 million are chronic carriers of the virus.^[1]^ HBV infection can develop into severe liver diseases, such as hepatic failure, cirrhosis, and hepatocellular carcinoma (HCC).^[2]^ The liver is a unique immune organ with many resident innate and adaptive immune cells, which induce various complex and sophisticated immune responses.^[3]^ Dysregulated immune response has been deemed to play a crucial role in the chronicity of an infection.^[4]^ The cellular immune response, especially the T cell immunologic response, is an important mechanism for the elimination and control of HBV infection.^[1,5]^ Additionally, T cells, which constitute the main cell population of specific immunity to eliminate and inhibit the virus, play an important role in anti-HBV infection-induced liver damage.^[6]^ As HBV enters the liver, many inflammatory cells, especially HBV-specific and nonspecific T cells, flow into the liver, and these can eliminate the virus by secreting cytokines or causing direct cell lysis.^[7,8]^ However, the above process can also cause apoptosis and necrosis of the infected hepatocytes, which is clinically manifested as an increase in transaminase levels and decrease and clearance of HBV DNA.^[9]^ During acute HBV infection, sufficient antiviral factors and functional T cells are produced to eliminate the virus.^[10]^ However, in CHB infection, the antiviral cytokines produced are not sufficient for viral clearance, and this further leads to T cell exhaustion and dysfunction.^[11]^ Inhibitory receptors play a key role in T cell exhaustion, which is an important factor influencing persistent HBV infection.^[12]^

Leukocyte-associated immunoglobulin-like receptor-1 (LAIR-1) is an inhibitory receptor expressed on various immune cells, similar to programmed cell death protein 1 (PD-1), cytotoxic T lymphocyte-associated protein 4 (CTLA4), and T cell immunoglobulin mucin-3 (TIM-3; first cloned in 1997).^[13]^ LAIR-1 is expressed on most human immune cells, including T cells, B cells, natural killer (NK) cells, monocytes, dendritic cells, basophils, eosinophils, and mast cells.^[13,14]^ In addition, LAIR-1 is expressed on CD34+ hematopoietic progenitor cells and most thymocytes.^[15,16]^ As a type I transmembrane glycoprotein, LAIR-1 contains extracellular single-chain immunoglobulin (Ig)-like domains and 2 immunoreceptor tyrosine-based inhibitory motifs (ITIMs) in the cytoplasmic region. Collagen, complement component C1q, and surfactant protein D are the ligands of LAIR-1. After the ligands are bound to LAIR-1, the ITIMs are phosphorylated and recruit phosphatases SHP-1, SHP-2, and C-terminal Src kinase to trigger a series of downstream events and eventually inhibit immune cell function.^[13]^ A recent study has shown that LAIR-1 inhibits T cell signaling by decreasing protein phosphorylation in the MAPK-associated signaling pathway.^[17]^

In vitro experiments showed that LAIR-1 can inhibit NK cell-mediated target cell lysis by both resting and activated NK cells.^[13,18]^ In T cells, LAIR-1 can inhibit the cytotoxic activity of effector T cells and activation of naïve T cells mediated by T cell receptors.^[19]^ LAIR-1 can inhibit the differentiation of peripheral blood precursors toward dendritic cells. Lastly, other studies reported that LAIR-1 can inhibit cytokine-mediated signals.^[13]^ Some researchers have observed LAIR-1 expression abnormalities in immune cells in chronic viral infections and autoimmune diseases, which suggests that LAIR-1 plays an important role in chronic inflammatory diseases. In chronic active Epstein–Barr virus disease and human immunodeficiency virus (HIV) infection, LAIR-1 expression on NK cells and B cells is decreased.^[20]^ Moreover, LAIR-1 is involved in the pathological process of inflammation in systemic lupus erythematosus and rheumatoid arthritis.^[21–23]^ Interestingly, some studies found that abnormal LAIR-1 expression in malignant tumors, including that in HCC,^[24]^ epithelial ovarian cancer,^[25]^ and oral squamous cell carcinoma, is related to prognosis.^[26]^ Furthermore, LAIR-1 plays an important role in tumor immune escape.^[27]^

Several studies focused on the expression of inhibitory receptors on immune cells to gain a comprehensive overview of T cell exhaustion in patients with CHB. However, whether the inhibitory receptor LAIR-1 plays an important role in T cell exhaustion in patients with CHB and LAIR-1 expression on T cells during HBV infection remains unknown. Therefore, in this study, we examined LAIR-1 expression frequency and levels on T cells and its 2 subsets (CD4+ and CD8+ T cells) to determine the correlation between LAIR-1 expression status in T cells and clinical and virological characteristics. Additionally, we discussed their differences at various disease stages in patients with CHB. We determined that LAIR-1 expression was related to CHB disease progression and thus can be a potential biomarker for evaluating inflammation and fibrosis.

## 2 Methods

### 2.1 Patients

From July 2015 to January 2017, we recruited 320 treatment-naïve patients with CHB from the Infectious Diseases Department Clinic at the Third Affiliated Hospital of Sun Yat-sen University, China. Exclusion criteria included patients who had previously received antiviral treatment (interferon or nucleoside/nucleotide analogs) and immunosuppressive therapy; were co-infected with HIV, hepatitis C virus, or hepatitis D virus; or had an autoimmune disease, fatty liver disease, cirrhosis, or malignancy. Patients were enrolled after fully understanding the study design and provided informed consent. The study was approved by the Institutional Review Board of the Third Affiliated Hospital of the Sun Yat-sen University, and all experiments were conducted as per the principles of the Declaration of Helsinki. Basic information, including the clinical and demographic data, of patients with CHB is shown in Table 1. Additionally, we collected peripheral blood samples from the participants for routine laboratory examinations and LAIR-1 testing. We recruited healthy controls (HCs), who showed normal liver functions and had no history of HBV infection as per the medical examination center of the Third Affiliated Hospital of Sun Yat-sen University. Based on laboratory test results, including biochemical, serologic, and virological parameters, according to the American Association for the Study of Liver Disease (AASLD) treatment guidelines, we categorized the participants into the following groups: immune tolerant (IT), immune active (IA), inactive CHB (IC), and gray zone (GZ). The classification criteria are presented in Table 2.

**Table 1:** Clinical–virological characteristics of patients included in the study.

Characteristics	IT (n = 31)	IA (n = 184)	IC (n = 48)	GZ (n = 57)	*P*
Age, y, median (quartile)	26 (24–31)	29.5 (25–34.75)	32 (28–39)	32 (27.5–38)	<.001
Sex					.428
Male, n (%)	19 (61.3%)	139 (75.5%)	35.3 (72.9%)	42 (73.7%)	
Female, n (%)	12 (38.7%)	45 (24.5%)	13 (27.1%)	15 (26.3%)	
BMI, median (quartile)	20.55 (18.38–22.58)	21.01 (19.26–22.76)	22.18 (20.3–23.39)	21.19 (19.5–23.28)	.058
AST, U/L, median (quartile)	25 (21–28)	61 (36–107.75)	24.5 (21.25–29)	26 (22–30.5)	<.001
ALT, U/L, median (quartile)	24 (19–29)	101 (55–172.25)	22.5 (17–29.75)	25 (20–32)	<.001
ALB, g/L, median (quartile)	44.8 (44–47.1)	44.8 (42.4–46.95)	46.8 (45.1–48.5)	46.65 (45.5–48.03)	<.001
GLB, g/L, median (quartile)	29.33 (26.35–31.5)	29 (26.42–32.21)	28.82 (27.06–31.2)	29.25 (26.73–31.39)	.933
TBIL, μmol/L, median (quartile)	13.05 (9.45–18.6)	15.7 (11.75–20.25)	12.2 (9.375–16.65)	12.05 (9.3,–5.65)	<.001
HBV DNA, Log IU/mL, median (quartile)	8.23 (8.23–8.23)	7.88 (6.18–8.23)	1.96 (1.36–3)	3.78 (3.24–4.48)	<.001
qHBsAg, IU/mL, median (quartile)	42428 (28177–52000)	10433.5 (2550.75–33712.75)	1031 (106–3701)	1278 (176.4–2615)	<.001
HBcAb					.002
>0.009, n (%)	24 (77.4%)	101 (54.9%)	38 (79.2%)	34 (59.6%)	
<0.009, n (%)	6 (19.4%)	82 (44.6%)	9 (18.8%)	23 (40.4%)	
Missing, n (%)	1 (3.2%)	1 (0.5%)	1 (2%)	0	
HBeAg status					<.001
Negative, n (%)	0 (0%)	42 (22.8%)	45 (93.8%)	48 (84.2%)	
Positive, n (%)	31 (100%)	142 (77.2%)	3 (6.3%)	9 (15.8%)	
APRI score, median (quartile)	0.2897 (0.2274–0.3732)	0.8824 (0.5696–1.684)	0.3034 (0.2395–0.3548)	0.2995 (0.2533–0.4448)	<.001

ALB = albumin, ALT = alanine aminotransferase, APRI = aspartate aminotransferase-to-platelet ratio index, AST = aspartate aminotransferase, BMI = body mass index, GLB = globulin, GZ = Gray zone, HBV = hepatitis B virus, IA = immune active, IC = inactive CHB, IT = immune tolerant, TBIL = total bilirubin.

**Table 2:** Disease phases classification criteria^2^.

Disease phases classification	ALT	HBV DNA	HBeAg
Immune active (IA)	Elevated	>20,000 IU/mL	Positive
		>2000 IU/mL	Negative
Inactive CHB (IC)	Normal	Low HBV DNA level	Negative
Immune tolerance (IT)	Normal	>1 million IU/mL	Positive
Gray zone (GZ)	Not classified as IC, IT, or IA		

Upper limit of normal (ULN) of ALT: 30 U/L for males and 19 U/L for females. ALT = alanine aminotransferase, HBV = hepatitis B virus.

### 2.2 Clinical and serological parameters

We tested hepatitis B surface antibody (HBsAb), hepatitis B e antigen (HBeAg), and hepatitis B core antibody (HBcAb) using commercial kits (Roche Diagnostics, Indianapolis, IN, USA) and quantitatively measured hepatitis B surface antigen (HBsAg) using the Elecsys HBsAg II Quant Reagent Kit (Roche Diagnostics) by following the manufacturer's instructions. We measured serum HBV DNA levels using Roche COBAS AmpliPrep/COBAS TaqMan HBV Test v2.0 (dynamic range: 20–1.7E+08 IU/mL; Roche Molecular Diagnostics, Branchburg, NJ, USA) by following the manufacturer's protocol. Guangzhou Supbio Bio-Technologies and Science Co., Ltd. (Guangzhou, China) performed the HBV pre-genomic RNA (pgRNA) level testing using a quantitative polymerase chain reaction. The reaction mixture was denatured at 95°C for 5 minutes, followed by 40 cycles at 95°C for 20 seconds and 60°C for 40 seconds. The APRI score was calculated as APRI = 100 (AST/upper limit of normal)/platelet count (10^9^ cells/L) for liver fibrosis evaluation.

### 2.3 Peripheral blood mononuclear cell isolation and flow cytometric analysis

We collected anticoagulated blood samples from all participants after obtaining informed consent. Peripheral blood mononuclear cells were obtained by density gradient centrifugation with Ficoll-Hypaque (Sigma, St Louis, MO) according to the manufacturer's protocol. We performed cell surface marker staining of the peripheral blood mononuclear cells using conjugated human antibodies, including FITC-CD56, PE-LAIR-1, PE-CF594-CD3, APC-CD4, and BV450-CD8 (eBioscience, San Diego, CA). Furthermore, we performed flow cytometry of the stained cells using Beckman Coulter Gallios Flow Cytometer (Beckman Coulter, Brea, CA) and analyzed the flow cytometry data using FlowJo V10 software (FlowJo LLC, Ashland, OR). The gating strategies are presented in Supplemental Figure 1.

### 2.4 Statistical analysis

Data that conformed to normal distribution are presented as mean ± standard deviation, and data that did not conform to normal distribution are presented as median (interquartile range). We determined the normal distribution using Shapiro–Wilk normality test. As most of the data did not conform to a normal distribution, Mann–Whitney *U* test was used to compare the differences between groups for a continuous variable. In addition, we performed an inter-group comparison of categorical variables using *χ*^2^ test. We also performed Spearman correlation analysis to analyze the correlation between LAIR-1 expression and clinical parameters. We implemented all statistical analyses and plotted graphs using IBM SPSS Statistics 24.0 (SPSS, IBM, NY) and Prism 8 (Graphpad, San Diego, CA). *P* < .05 indicated a significant difference.

## 3 Results

### 3.1 Characteristics and LAIR-1 expression on T cells in patients with CHB

**Figure 1:**
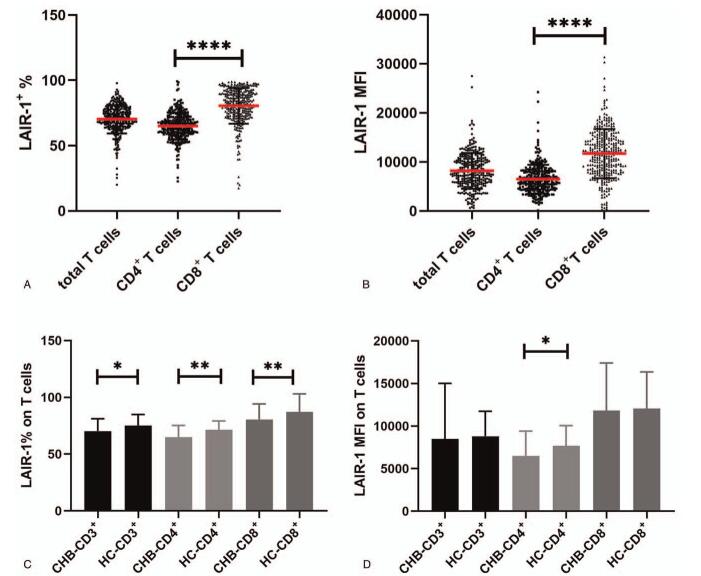
Leukocyte-associated immunoglobulin-like receptor-1 (LAIR-1) expression on T cells and its subsets in treatment-naïve patients with chronic hepatitis B (CHB) and healthy controls (HCs). (A) The frequency of LAIR-1 expression on CD3+, CD4+, and CD8+ T cells in patients with CHB. (B) The median fluorescence intensity (MFI) of LAIR-1 on CD3+, CD4+, and CD8+ T cells in patients with CHB. (C) The frequency of LAIR-1 expression on CD3+, CD4+, and CD8+ T cells in patients with CHB and HCs. (D) The MFI of LAIR-1 on CD3+, CD4+, and CD8+ T cells in patients with CHB and in HCs. Group differences were analyzed using Mann–Whitney *U* test. ^∗^*P* < .05; ^∗∗^*P* < .01; ^∗∗∗^*P* < .001; ^∗∗∗∗^*P* < .0001.

According to the inclusion and exclusion criteria, 320 patients with CHB in different phases were enrolled and categorized into four groups (IA = 184, IT = 31, IC = 48, and GZ = 57) according to their laboratory test results. Patient clinical features are presented in Table 1. First, we detected LAIR-1 expression on T cells and the two T cell subsets (CD4+ T cells and CD8+ T cells) to gain a preliminary understanding of LAIR-1 expression on T cells. We observed that both the expression frequency and mean fluorescence intensity (MFI) of LAIR-1 was significantly higher in CD8+ T cells than in CD4+ T cells (frequency: 82.4% vs 65.15% and MFI: 11,767 vs 6167, respectively; *P* < .0001; Fig. 1A–B). The expression frequency of LAIR-1 on total T cells, CD4+ T cells, and CD8+ T cells of patients with CHB was significantly lower than that in the HC group (70.8 vs 78.5, *P* = .0106; 65.15 vs 74.3, *P* = .0019; 82.4 vs 92.1, *P* = .0019, respectively; Fig. 1C). However, a significantly lower MFI of LAIR-1 in patients with CHB than that in HC group was observed only in case of CD4+ T cells (6167 vs 7401, *P* = .0251; Fig. 1D).

### 3.2 Expression of LAIR-1 on T cells in patients with CHB in different disease phases

**Figure 2:**
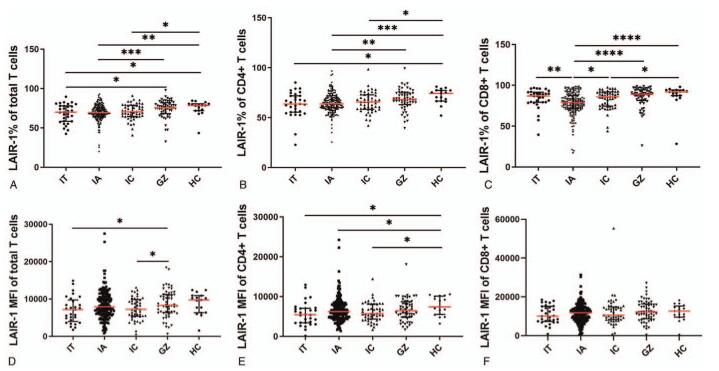
Frequency and mean fluorescent intensity (MFI) of leukocyte-associated immunoglobulin-like receptor-1 (LAIR-1) on T cells and its subset cells in treatment-naïve patients with chronic hepatitis B (CHB) in different disease phases and healthy controls (HCs). (A–C) The expression frequency of LAIR-1 on T cells and subset cells in HCs and patients with CHB in immune tolerant (IT), immune active (IA), inactive carrier (IC), and gray zone (GZ) phases. (D–F) The MFI of LAIR-1 on T cells and subset cells in HCs and IT, IA, IC, and GZ phases of patients with CHB. Group differences were analyzed using Mann–Whitney *U* test. ^∗^*P* < .05, ^∗∗^*P* < .01, ^∗∗∗^*P* < .001, ^∗∗∗^*P* < .0001.

To investigate the differential expression of LAIR-1 on peripheral blood T cells in patients with CHB in different disease phases, we characterized the expression frequency and MFI of LAIR-1 on total T cells, CD4+ T cells, and CD8+ T cells in our patient cohort. The expression frequency and MFI of LAIR-1 on T cells and the 2 T cell subsets in the five groups, including the HC group, are shown in Figure 2. The results showed significant differences in the expression frequency of LAIR-1 on total T cells, CD4+ T cells, and CD8+ T cells within some groups (Fig. 2A–C). For instance, we observed significant differences in the expression frequency of LAIR-1 on total T cells, CD4+ T cells, and CD8+ T cells between the IA and GZ, IA and HC, and IC and HC groups (*P* < .05). The expression frequency of LAIR-1 on each of the T cell subsets was the highest in the GZ group and lowest in the IA group. Additionally, in some groups, there were significant differences in the MFI of LAIR-1 on total T cells and CD4^+^ T cells but not in CD8+ T cells (Fig. 2D–F). A significantly lower MFI of LAIR-1 on total T cells was observed in the IC and IT groups than in the GZ group (*P* = .0475 and *P* = .0455, respectively; Fig. 2D). We observed upregulated LAIR-1 expression in the IT, IA, and IC groups than in the HC group (*P* = .0202, *P* = .0408, and *P* = .0501, respectively; Fig. 2E). However, no significant differences were observed in the MFI of LAIR-1 on CD8+ T cells between the groups (Fig. 2F).

### 3.3 Correlation between the expression of LAIR-1 and virological indicators

**Figure 3:**
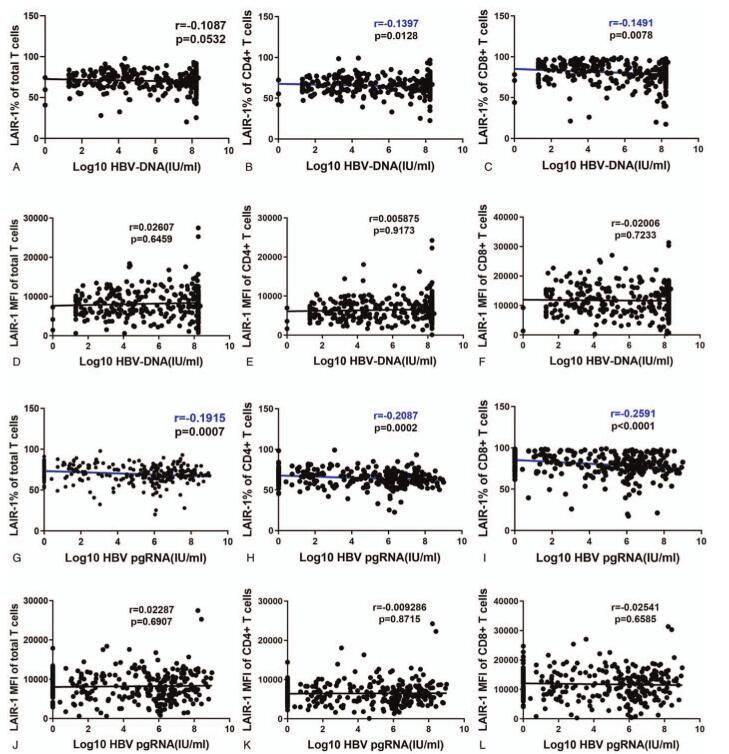
Correlation between leukocyte-associated immunoglobulin-like receptor-1 (LAIR-1) expression and serum virological indicators. (A-C) Correlations between HBV DNA levels and frequency of LAIR-1 expression on CD3+, CD4+, and CD8+ T cells. (D-F) Correlations between HBV DNA levels and LAIR-1 MFI on CD3+, CD4+, and CD8+ T cells. (G–I) Correlations between HBV pgRNA levels and LAIR-1 expression frequency on CD3+, CD4+, and CD8+T cells. (J-L) Correlations between HBV pgRNA levels and LAIR-1 MFI on CD3+, CD4+, and CD8+ T cells. Spearman's correlation tests were performed for correlation analysis. HBV = hepatitis B virus, pgRNA = pregenomic RNA.

Because HBV virological characteristics are important indicators for patient grouping, we analyzed the correlation between LAIR-1 expression on T cells and levels of serum HBV virological characteristics, including HBV DNA and pgRNA levels. LAIR-1 expression frequency on total T cells, CD4+ T cells, and CD8+ T cells showed a significant negative correlation with HBV pgRNA level (*r* = −0.1915, *P* = .0007; *r* = −0.2087, *P* = .0002; and *r* = −0.2591, *P* < .0001, respectively; Fig. 3A–C). We observed a significant negative correlation between serum HBV DNA level and LAIR-1 expression frequency on CD4+ T cells (*r* = −0.1397, *P* = .0128) and CD8+ T cells (*r* = −0.1491, *P* = .0078) but not on total T cells (*P* > .05; Figure 3G–I). However, no significant correlation was observed between MFI of LAIR-1 on T cells and the 2 subsets and serum HBV DNA level (Fig. 3D–F, J–L).

### 3.4 Correlation between the expression of LAIR-1 and HBV antigens and antibodies

**Figure 4:**
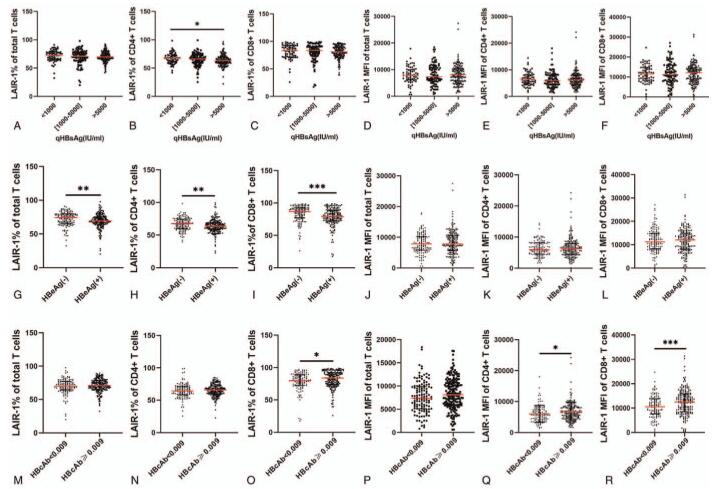
Correlation between leukocyte-associated immunoglobulin-like receptor-1 (LAIR-1) expression in patients with chronic hepatitis B (CHB) and hepatitis B virus (HBV) antigen levels. First, patients with CHB were classified into 3 groups based on their HBsAg levels: <1000, 1000∼5000, and >5000 groups. Differences in LAIR-1 (A–C) expression frequency and (D–F) mean fluorescent intensity (MFI) on CD3+, CD4+, and CD8+ T cells in the groups that were analyzed. Differences in LAIR-1 (G–I) expression frequency and (J–L) MFI on CD3+, CD4+, and CD8+ T cells between HBeAg-positive and HBeAg-negative patients with CHB. Lastly, the patients with CHB were categorized into 2 groups based on their HBcAb levels (HBcAb ≥0.009 and HBcAb <0.009). Differences in LAIR-1 (M–O) expression frequency and (P–R) MFI on CD3+, CD4+, and CD8+ T cells between the groups were analyzed. Differences were analyzed using Mann–Whitney *U* test. HBcAb = hepatitis B core antibody, HBeAg = hepatitis B e antigen, HBsAg = hepatitis B surface antigen. ^∗^*P* < .05, ^∗∗^*P* < .01, ^∗∗∗^*P* < .001, ^∗∗∗∗^*P* < .0001.

HBV antigens including HBsAg, HBsAb, HBeAg, and HBcAb were analyzed in this study (Fig. 4). First, we analyzed the association between LAIR-1 expression and quantitative HBsAg level. We categorized the patients into three groups according to the HBsAg levels: <1000, (1000,5000), and >5000 IU/mL groups. We observed a significant difference in the LAIR-1 expression frequency on the CD4+ T cells between the HBsAg <1000 IU/mL and HBsAg >5000 IU/mL groups (67.8% vs 63.4%, *P* = .0175; Fig. 4B). No significant difference was observed in the expression of LAIR-1 between HBsAb-positive and HBsAb-negative patients (data not shown). The frequency of LAIR-1 expression on total T cells, CD4+ T cells, and CD8+ T cells were significantly higher in the HBeAg-negative patients than in the HBeAg-positive patients with CHB (*P* = .0040, *P* = .0018, and *P* = .0002, respectively; Fig. 4G–H). Next, we classified the patients into 2 groups according to their HBcAb levels (HBcAb <0.009 and HBcAb ≥0.009). In our approach, HBcAb detected values ≤1 Cut of Index (COI) were considered positive. For positive results, higher the detection value, lower the HBcAb level. LAIR-1 expression frequency on CD8+ T cells was significantly higher in the HBcAb ≥0.009 group than the HBcAb <0.009 group (*P* = .0216; Fig. 4O). Moreover, we observed a significantly higher LAIR-1 MFI on CD4+ T cells and CD8+ T cells in the HBcAb ≥0.009 group than in the HBcAb < 0.009 group (*P* = .0176 and *P* = .0010, respectively; Fig. 4Q–R).

### 3.5 Correlation between the expression of LAIR-1 and liver function indicators

**Figure 5:**
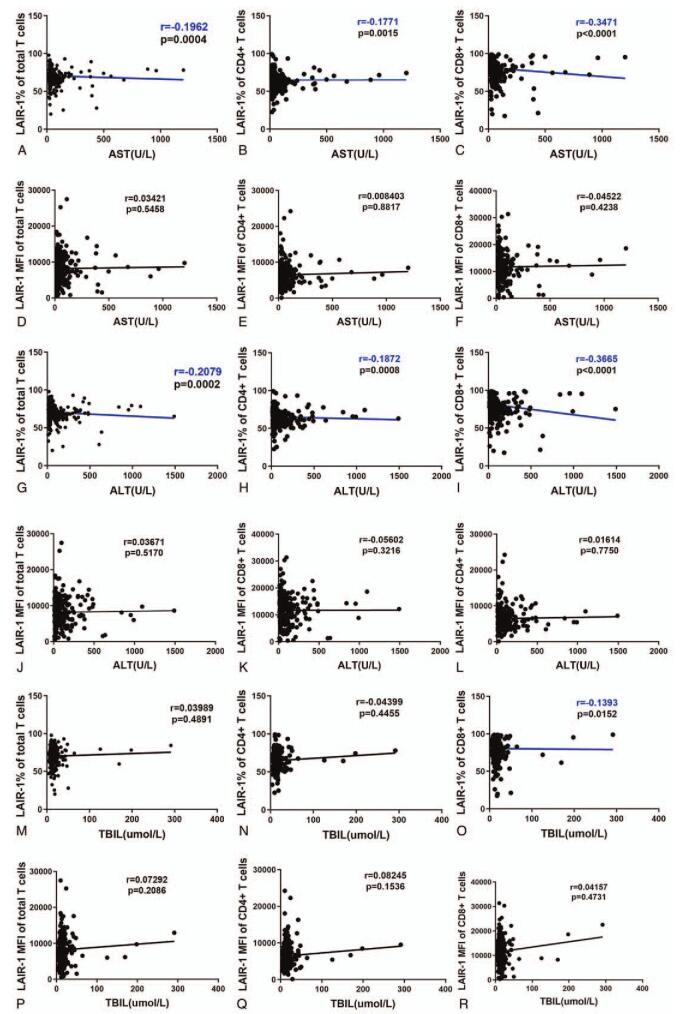
Correlation between leukocyte-associated immunoglobulin-like receptor-1 (LAIR-1) expression and liver inflammation indicators. Correlations between aspartate aminotransferase (AST) level and LAIR-1 expression frequency (A–C) and AST level and mean fluorescent intensity (MFI) of LAIR-1 (D–F) on CD3+, CD4+, and CD8+ T cells. Correlations between alanine aminotransferase (ALT) level and LAIR-1 expression frequency (G–I) and ALT level and LAIR-1 MFI (J–L) on CD3+, CD4+, and CD8+ T cells. Correlations between total bilirubin (TBIL) level and LAIR-1 expression frequency (M–O) and TBIL level and LAIR-1 MFI (P–R) on CD3+, CD4+, and CD8+ T cells. Spearman correlation tests were performed for the correlation analysis.

To understand the correlation between the expression of LAIR-1 and liver function indicators, such as alanine aminotransferase (ALT), aspartate aminotransferase (AST), and total bilirubin (TBIL), we performed a correlation analysis. We observed a significant negative correlation between AST level and LAIR-1 expression frequency on total T cells (*r* = −0.1962, *P* = .0004), CD4+ T cells (*r* = −0.1771, *P* = .0015), and CD8+ T cells (*r* = −0.3471, *P* < .0001; Fig. 5A–C). The correlation between ALT level and LAIR-1 expression frequency on total T cells, CD4+ T cells, and CD8+ T cells was the same as that between AST level and LAIR-1 expression frequency on these cells (*r* = −0.2079, *P* = .0002; *r* = −0.1872, *P* = .0008; *r* = −0.3665, *P* < .0001, respectively; Fig. 5G–I). However, no significant correlation was observed between AST and ALT levels and MFI of LAIR-1 on T cells and its subsets (Figure 5D-F, J-L). Lastly, a significant but weak negative correlation was observed between TBIL and LAIR-1 expression frequency on CD8+ T cells (*r* = −0.1393, *P* = .0152; Fig. 5O).

### 3.6 Correlation between the expression of LAIR-1 and fibrosis

**Figure 6:**
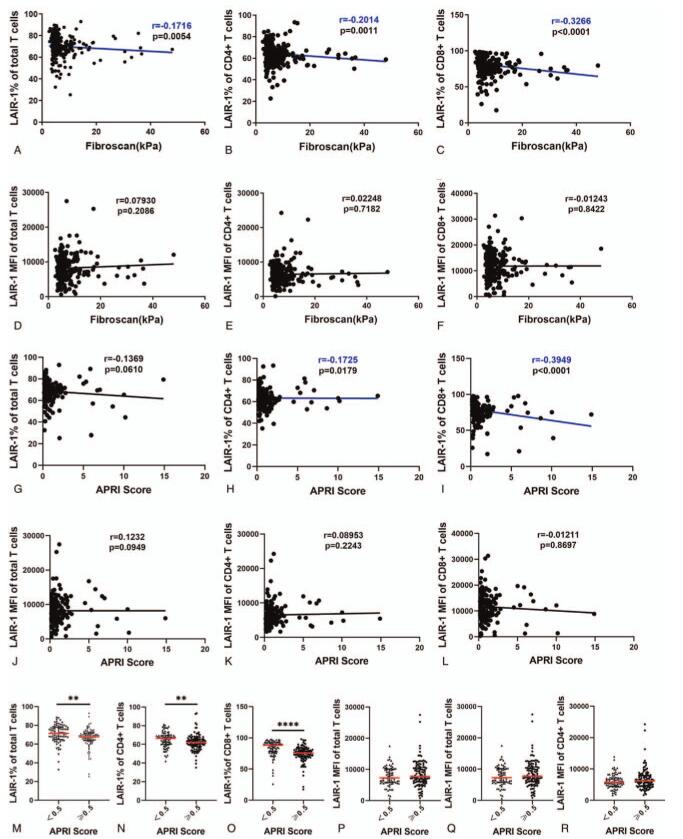
Correlation of leukocyte-associated immunoglobulin-like receptor-1 (LAIR-1) expression with fibrosis. Correlations between FibroScan value and LAIR-1 expression frequency (A–C) and FibroScan value and LAIR-1 mean fluorescent intensity (MFI) (D-F) on CD3+, CD4+, and CD8+ T cells. Correlations between APRI score and LAIR-1 expression frequency (G–I) and APRI score and LAIR-1 MFI (J–L) on CD3+, CD4+, and CD8+ T cells. Spearman correlation tests were performed for correlation analysis. APRI = aspartate aminotransferase-to-platelet ratio index, AST = aspartate aminotransferase $APRI\;score=\frac{AST+ULN\times 100}{PLT\left(\frac{10^{9}}{L}\right)}$, where, ULN is upper limit of normal value and PLT indicates platelet counts.

As extracellular matrix (ECM) collagen is a ligand of LAIR-1, the excessive deposition of which occurs in tissues in hepatic fibrosis, we analyzed the correlation between fibrosis markers (FibroScan value and AST-to-platelet ratio index [APRI] score) and LAIR-1 expression on T cells in the patients. There was a significant negative correlation between the FibroScan value and LAIR-1 expression frequency on total T cells (r = -0.1716, p = .0054), CD4+ T cells (r = -0.2014, p = .0011), and CD8+ T cells (r = -0.3266, p < .0001; Figure 6A–C). In addition, we observed a significant negative correlation between the APRI score and LAIR-1 expression frequency on CD4+ T cells (r = -0.1725, p = .0179) and CD8+ T cells (r = -0.3949, p < .0001; Figure 6H–I). Next, we categorized the patients into two groups: significant fibrosis group and non-significant fibrosis group (APRI cutoff value = 0.5) according to the World Health Organization (WHO) “Guidelines for the Prevention, Care and Treatment of Persons with Chronic Hepatitis B Infection” and found that LAIR-1 expression frequency on total T cells, CD4+ T cells, and CD8+ T cells in the non-significant fibrosis group was significantly higher than that in the significant fibrosis group (p = .0032, p = .0020, and p < .0001, respectively; Figure 6M–O).

## 4 Discussion

T cells play a crucial role in the pathogenesis of CHB.^[28,29]^ The balance between the inhibitory and activated receptors on immune cells plays an important role in maintaining the immune functions.^[30]^ At present, few studies have reported that the decrease in the frequency and function of HBV-specific T cells is a reason for the difficulty in HBV-specific clearance and treatment.^[28,31]^ CHB with persistent HBV infection and active HBV replication are important risk factors for the progression of cirrhosis and HCC.^[32]^ Sustained high levels of HBV replication can lead to repeated liver necrosis and inflammation, ultimately leading to fibrosis, cirrhosis, and HCC.^31^ In addition, the dysregulation of activation/homeostasis of the immune system can lead to the progression to cirrhosis and HCC. Normally, inhibitory receptors on immune cells prevent the immune cells from over-activating and causing tissue damage. However, during viral infections or in tumors, the inhibitory receptors are highly expressed, leading to immune cell exhaustion.^[33]^ At present, studies on inhibitory receptors in patients with CHB are mainly focused on PD-1, CTLA4, and TIM-3^[34]^; however, studies on the cell expression of LAIR-1 are scarce.

In this study, we examined LAIR-1 expression in peripheral blood T cells acquired from patients with CHB to determine if they may be involved in immunoregulation in CHB. The results showed that LAIR-1 was expressed in most T cells; however, the frequency of cells expressing LAIR-1 and the expression levels of LAIR-1 were varied. For instance, a higher LAIR-1 expression frequency was observed in CD8+ T cells than in CD4+ T cells, suggesting that the expression mechanism of LAIR-1 in T cells is complex. Further, the frequency of T cells expressing LAIR-1 and the expression level of LAIR-1 on T cells were lower in patients with CHB than in the HCs. Moreover, we observed some variance in LAIR-1 expression frequency and MFI on total T cells, CD4+ T cells, and CD8+ T cells based on the different CHB disease stages. Our data showed that LAIR-1 expression frequency on both total T cells and T cell subsets in IA patients was the lowest of that among all CHB stages, indicating that the inflammatory/immune microenvironment induced by HBV infection could influence the expression of LAIR-1. IA patients are usually considered immune active and are associated with a higher level of ALT, HBV-DNA, and serious inflammation. T cell activation in vitro can downregulate LAIR-1 and is parallel with an increased activation level of T cells.^[20]^ HBV can cause inflammation in the liver that is mediated by host immune responses to the hepatocytes infected by HBV as a non-cytopathic virus. HBV activates the CD4+ and CD8+ T cells and then causes a cytokine release, which results in the downregulation of LAIR-1 expression.^[20]^

Previous studies showed that LAIR-1 is upregulated in naïve immune cells than in mature immune cells, suggesting that T cells in patients with CHB are active and mature. Indeed, T cells in patients with CHB are exhausted, which is characterized by low proliferative ability, decreased antiviral cytokine production, and suppressed cytotoxicity. One possible explanation is that other inhibitory receptors play a major role in T cell exhaustion during HBV infection, whereas LAIR-1 expressed on T cells is shed at the early stage of HBV infection. Jansen et al. found that soluble LAIR-1 can be detected in stimulated lymphocyte culture supernatant, suggesting that LAIR-1 falls off from the membranes upon stimulation. In addition, after in vitro stimulation, LAIR-1 was present in intracellular stores, leading to a decrease in LAIR-1 expression on T cell surface.^[20]^ However, the exact mechanism of LAIR-1 in HBV infection remains unclear.

During HBV infection, LAIR-1 and its ligands may be involved in regulating the immune response, and their interaction sets a “threshold” for the activation of the immune cells. When the activation signal (for instance, causative agents, antigen-presenting cells, and cytokines) is greater than the “threshold,” the T cells are activated. At the same time, by downregulating the expression of LAIR-1, the “threshold” is reduced, which is conducive to the activation of the T cells. Our results showed that LAIR-1 was highly expressed on the CD8+ T cells in the IT patients, which suggested a low activation status and high activation threshold of the CD8+ T cells, leading to immune tolerance.

Notably, LAIR-1 was highly expressed on the T cells and their subsets in the GZ patients. According to the AASLD guidelines for the treatment of CHB, patients with CHB presenting mild ALT elevation (1–2 × ULN) and low level of HBV DNA (<20,000 IU/mL for HBeAg-positive patients and <2000 IU/mL for HBeAg-negative patients) were classified as GZ^[2]^ because we could not classify them as IT, IA, or IC based on their serum virological and biochemical characteristics. For the GZ patients, follow-up of ALT and HBV DNA levels and/or liver biopsy can help in determining if treatment is necessary. Li et al. found that the cytolytic capacity of NK cells in GZ is higher than that in the HCs and IA patients.^[31]^ Previous studies demonstrated that a sizable fraction of the GZ patients (9%) can show progression to cirrhosis; therefore, early initiation of treatment is necessary to induce HBV immune control and avoid poor prognosis.^[35]^ High expression of LAIR-1 in GZ patients suggests that T cells are at a lower activation level and may be difficult to activate, owing to which the antiviral effects in the GZ patients are usually not satisfactory. Based on the above results, LAIR-1 could be a treatment target to control inflammation and improve antiviral effects.

In contrast, to the best of our knowledge, our study is the first to elucidate a detailed relationship between LAIR-1 expression level and liver inflammation, fibrosis, and serum HBV virological indexes in a cohort of patients with CHB. We found that LAIR-1 expression on both the total T cells and two T cell subsets was negatively correlated with liver inflammation (ALT, AST, and TBIL levels), serum HBV virological indexes (HBV DNA and HBV pgRNA levels), and fibrosis (FibroScan value and APRI score). Moreover, LAIR-1 expression on T cells was related to the HBeAg state, wherein patients with CHB showing a positive HBeAg had a lower LAIR-1 expression on the T cells. Previous studies showed that high levels of HBcAb are related to a strong adaptive antiviral immune response during HBV infection. In fact, HBcAb can be a predictor of antiviral efficacy.^[36–39]^ These aspects could account for our finding that patients with higher HBcAb levels had a lower LAIR-1 level, which suggests a stronger immune response. CHB stages according to the AASLD guidelines for hepatitis B treatment are mainly based on ALT and HBV DNA levels and the HBeAg state in patients. These results are consistent with those of LAIR-1 expression in different patients with CHB, thereby suggesting that LAIR-1 plays a critical role during HBV infection.

The relevance of LAIR-1 in liver disease is that one of its major ligands is collagen. A characteristic feature of liver fibrosis is the activation of hepatic stellate cells and increase in collagen deposition.^[40–41]^ Because we did not perform liver biopsy in patients with CHB, we calculated the APRI score and accordingly classified the patients into two groups to understand the correlation between the degree of liver fibrosis and expression of LAIR-1. WHO guidelines recommend an APRI score of 0.5 as a cutoff value to distinguish between significant and non-significant fibrosis. Our results indicated that LAIR-1 expression frequency on T cells and its subsets was lower in the significant fibrosis group than in the non-significant fibrosis group. Thus, we hypothesized that collagen was highly expressed in the fibrotic tissues, which then downregulated LAIR-1 expression. Consistent with our results, María et al also found decreased LAIR-1 expression frequency on macrophages in patients with liver cirrhosis.^[42]^

However, Kennedy et al determined LAIR-1 expression on global T cells but found no significant difference in LAIR-1 expression on CD4+ and CD8+ T cells between patients with CHB and HCs.^[43]^ Hansi et al studied LAIR-1 expression in patients with CHB and found circulating NK cells with higher frequencies of LAIR-1-expressing NK cells in patients with CHB than in HCs (N. K. H, MD, unpublished data, 2017). Their conclusions were contradictory to ours; this is probably because of the different number and baseline characteristics of the selected subjects. At present, characteristic LAIR-1 expression on T cells and NK cells in patients with CHB remains unclear and thus requires further study.

The expression pattern of other inhibitory receptors, such as PD-1, TIM-3, and CTLA4, differed from that of LAIR-1 in patients with CHB. PD-1, CTLA4, and TIM-3 on T cells are upregulated in chronic HBV infection.^[44–46]^ As a ligand of LAIR-1, collagen is an abundant ECM protein, and this emphasizes new immunomodulatory effects of ECM. On the contrary, most ligands of the inhibitory receptors of immune cells are cell bound and play a role via the interaction between cells. Taken together, LAIR-1 is a distinct immune inhibitory receptor that is relative to others.

The limitations of the present study must be acknowledged. We did not examine LAIR-1 expression in liver tissues because of a lack of samples. Further, we did not include patients with liver cirrhosis and HCC in this study. Therefore, we were unable to observe changes in LAIR-1 expression in the sustained progression of “CHB–cirrhosis–HCC,” although it is of keen interest. For LAIR-1, the mechanism associated with CHB disease states is still undetermined, and further research is required to better understand the underlying mechanisms. As a ligand of LAIR-1, collagen is abundant in the ECM, which suggests that the ECM proteins may play an important role in immune regulation.

Thus, the findings of this study will help us better understand the role of inhibitory receptors on T cells in patients with CHB. Similar to other inhibitory receptors, such as PD-1 and CTLA4, manipulating LAIR-1 may be a strategy for regulating inflammation and disease activity in CHB.

## Acknowledgments

The authors thank Editage (www.editage.com) for English language editing.

## Author contributions

**Conceptualization:** Yuehua Huang.

**Data curation:** Yanhua Bi.

**Funding acquisition:** Yuehua Huang, Yurong Gu.

**Investigation:** Yurong Gu, Yanhua Bi, Huan Wei, Chunhong Liao.

**Methodology:** Huan Wei.

**Resources:** Yurong Gu.

**Validation:** Yurong Gu, Zexuan Huang, Weixin Liao.

**Visualization:** Yanhua Bi, Jing Li.

**Writing – original draft:** Yurong Gu, Yanhua Bi.

**Writing – review & editing:** Yuehua Huang.

## Supplementary Material

Supplemental Digital Content
