# Supplementary figures and images for: Expression and clinical significance of inhibitory receptor Leukocyte-associated immunoglobulin-like receptor-1 on peripheral blood T cells of chronic hepatitis B patients: A cross-sectional study
Source: Medicine (Baltimore). 2021 Jul 23;100(29):e26667. doi: 10.1097/MD.0000000000026667 (PMC8294879; doi:10.1097/MD.0000000000026667)

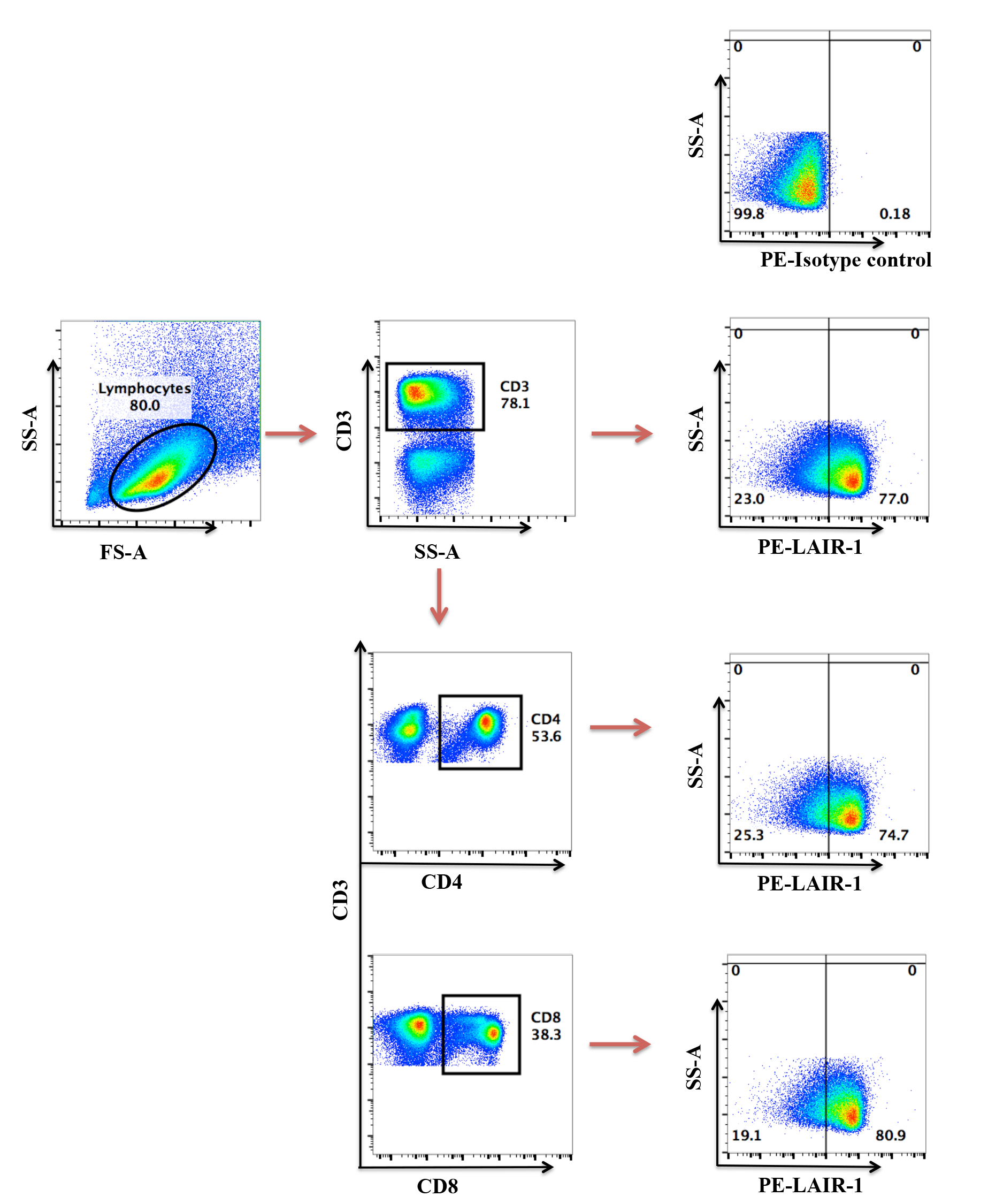

Supplement: Supplemental Digital Content [file medi-100-e26667-s001.tif]
